# Dietary amino acids, macronutrients, vaginal birth, and breastfeeding are associated with the vaginal microbiome in early pregnancy

**DOI:** 10.1128/spectrum.01130-24

**Published:** 2024-10-04

**Authors:** Gillian A. Corbett, Rebecca Moore, Conor Feehily, Sarah Louise Killeen, Eileen O'Brien, Douwe Van Sinderen, Elizabeth Matthews, Roisin O'Flaherty, Pauline M. Rudd, Radka Saldova, Calum J. Walsh, Elaine M. Lawton, David A. MacIntyre, Siobhan Corcoran, Paul D. Cotter, Fionnuala M. McAuliffe

**Affiliations:** 1 UCD Perinatal Research Centre, UCD School of Medicine, University College Dublin, Dublin, Ireland; 2 National Maternity Hospital, Dublin 2, Ireland; 3 School of Infection and Immunity, University of Glasgow, Glasgow, United Kingdom; 4 School of Biological, Health and Sports Sciences, Technological University Dublin, Dublin, Ireland; 5 School of Microbiology, University College Cork, Cork, Ireland; 6 APC Microbiome Ireland, University College Cork, Cork, Ireland; 7 GlycoScience Group, National Institute for Bioprocessing Research and Training (NIBRT), Dublin, Ireland; 8 Department of Chemistry, Maynooth University, Maynooth, Ireland; 9 Bioprocessing Technology Institute, AStar, Singapore, Singapore; 10 College of Health and Agricultural Science (CHAS), UCD School of Medicine, University College Dublin, Dublin, Ireland; 11 Teagasc Food Research Centre, Moorepark, Cork, Ireland; 12 The Centre for Pathogen Genomics, Department of Microbiology & Immunology, Doherty Institute for Infection & Immunity, University of Melbourne, Melbourne, Victoria, Australia; 13 Division of the Institute of Reproductive and Developmental Biology, Department of Metabolism, Digestion, and Reproduction, March of Dimes Prematurity Research Centre, Imperial College London, London, United Kingdom; Chengdu University, Chengdu, Sichuan, China

**Keywords:** vagina, microbiota, pregnancy, metagenomic sequencing, beta diversity, alpha diversity, community state type, environmental, diet, exercise

## Abstract

The vaginal microbiome is a key player in the etiology of spontaneous preterm birth. This study aimed to illustrate maternal environmental factors associated with vaginal microbiota composition and function in pregnancy. Women in healthy pregnancy had vaginal microbial sampling from the posterior vaginal fornix performed at 16 weeks gestation. After shotgun metagenomic sequencing, heatmaps of relative abundance data were generated. Community state type (CST) was assigned, and alpha diversity was calculated. Demography, obstetric history, well-being, exercise, and diet using food frequency questionnaires were collected and compared against microbial parameters. A total of 119 pregnant participants had vaginal metagenomic sequencing performed. Factors with strongest association with beta diversity were dietary lysine (adj-*R*^2^ 0.113, *P* = 0.002), valine (adj-*R*^2^ 0.096, *P* = 0.004), leucine (adj-*R*^2^ 0.086, *P* = 0.003), and phenylalanine (adj-*R*^2^ 0.085, *P* = 0.005, Fig. 2D). Previous vaginal delivery and breastfeeding were associated with vaginal beta diversity (adj-*R*^2^ 0.048, *P* = 0.003; adj-*R*^2^ 0.045, *P* = 0.004), accounting for 8.5% of taxonomy variation on redundancy analysis. Dietary fat, starch, and maltose were positively correlated with alpha diversity (fat +0.002 SD/g, *P* = 0.025; starch +0.002 SD/g, *P* = 0.043; maltose +0.440 SD/g, *P* = 0.013), particularly in secretor-positive women. Functional signature was associated with CST, maternal smoking, and dietary phenylalanine, accounting for 8.9%–11% of the variation in vaginal microbiome functional signature. Dietary amino acids, previous vaginal delivery, and breastfeeding history were associated with vaginal beta diversity. Functional signature of the vaginal microbiome differed with community state type, smoking, dietary phenylalanine, and vitamin K. Increased alpha diversity correlated with dietary fat and starch. These data provide a novel snapshot into the associations between maternal environment, nutrition, and the vaginal microbiome.

**IMPORTANCE:**

This secondary analysis of the MicrobeMom randomized controlled trial reveals that dietary amino acids, macronutrients, previous vaginal birth, and breastfeeding have the strongest associations with vaginal taxonomy in early pregnancy. Function of the vaginal niche is associated mainly by species composition, but smoking, vitamin K, and phenylalanine also play a role. These associations provide an intriguing and novel insight into the association between host factors and diet on the vaginal microbiome in pregnancy and highlight the need for further investigation into the complex interactions between the diet, human gut, and vaginal microbiome.

## INTRODUCTION

The vaginal microbiome is an integral component of the human microbiome and plays a fundamental role in fertility and pregnancy health. The human microbiome impacts human health and disease states via mediation of local and systemic immune responses (1). Interaction between microbiota and host gut, immune and neuro-endocrine systems is associated with a broad variety of complex chronic disease, including immune dysfunction (asthma and allergy) (2, 3), autoimmune diseases, including inflammatory bowel disease (4, 5), metabolic dysfunction, and sinister processes such as malignancy (4, 6). In pregnancy, host response to specific vaginal microbiota is linked with adverse outcomes, such as miscarriage, cervical shortening, and preterm birth (7–10). It is now well established that in healthy pregnancy, the vaginal microbiome stabilizes as a *Lactobacillu*s spp.-dominant ecosystem, where *Lactobacillus crispatus* is often the most dominant species in Caucasian populations (11, 12). As reported in recent meta-analyses, risk of adverse pregnancy outcomes, such as preterm birth, is highest in groups with low *Lactobacillus* spp. dominance and high alpha diversity, such as community state type (CST) IV (13, 14). This complements previous data linking low vaginal *Lactobacillus* spp. dominance with proinflammatory states and adverse pregnancy outcomes, such as bacterial vaginosis, prelabor preterm rupture of membranes, and preterm birth (9).

Regarding the role of the vaginal microbiome in shaping reproductive outcomes, the influence of demographic and geo-ethnical factors on compositional structure of the niche is of increasing importance. For example, parity has recently been associated with vaginal microbial composition, and nulliparous women are significantly more likely to harbor *Lactobacillus* spp. dominance compared to their multiparous counterparts (12). Vaginal taxonomy also varies with menstrual stage, sexual activity, and pregnancy itself (11, 15). Ethnicity is strongly linked to vaginal microbiota, with lower rates of *L. crispatus* abundance and higher alpha diversity observed in African American and Indian populations (16–18). Socio-economic factors, such as lower education status, have also been linked to suboptimal microbial parameters in pregnancy (12, 19), likely due, in part, to variations in diet, obesity, smoking, hygiene practices, or general lifestyle factors (16, 20). Dietary intake is a pivotal environmental factor for a myriad of health parameters, including obesity, metabolic profile, and immunological conditions, and is linked to gut microbial health and function (21–24). The gut microbiome is believed to play an integral regulatory role on many health systems, including the gut-brain axis and hormonal immune-endocrine pathways (25, 26). This regulation is actioned by short-chain fatty acids (SCFAs), propionate, butyrate, and acetate (25, 26). SCFAs are the microbial fermentation end-products of resistant starch, fiber, and non-starch polysaccharides ingested in the diet (27–29), while additional SCFAs are generated in the distal colon from fermentation of amino acids (AA) (30, 31). Although the regulatory role of gut microbiota on vaginal taxonomy and function is hypothesized, the interplay between these niches is yet to be fully understood. Environment and taxonomy only capture part of the host-microbe interaction, and there is a dearth of data on the interplay between maternal factors and the functional signature of the vaginal microbiome.

Another factor that is involved in the host-microbe interaction is secretor status. Secretor status relates to the secretion of blood group antigens into maternal body fluids and is controlled by the FUT2 gene and enzyme activity, with approximately 80% of a general population being secretor positive (32). Secretor-positive women produce the glycan H-antigen, after FUT2 enzyme adds fucose to a glycoprotein (33, 34). The H-antigen is expressed on mucosal surface where they may influence infection susceptibility via interactions between pathogen and host (35). “Non-secretor” status has been linked with various adverse health outcomes, including inflammatory bowel disease, autoimmune conditions, and type 1 diabetes (36). In pregnancy, secretor-negative status has been linked with shorter gestational age at delivery, particularly in lactobacillus deplete vaginal environments (37), but the mechanism underlying this link is not yet understood. It is also not yet known how secretor status interacts with environment and vaginal microbiome.

The aim of this study was to examine the composition and function of vaginal microbiota in a healthy pregnancy cohort and explore the interplay of these in early pregnancy with maternal factors, including demographic and socioeconomic factors, exercise patterns, maternal well-being, and daily dietary habits.

## MATERIALS AND METHODS

### Study design and participants

This study is a secondary analysis of the MicrobeMom randomized controlled trial (38). The MicrobeMom trial [ISRCTN53023014 (39)] examined the ability of an orally administered *Bifidobacterium breve* strain to transfer from maternal gut to neonatal gut. The individuals recruited were healthy women in early pregnancy. Inclusion criteria for enrollment had a body mass index (BMI) of 18.5–35 kg/m^2^ and were of age over 18 years with adequate English and ability to give informed consent. Individuals were excluded if they had a history of diabetes mellitus, pre-diabetes, gestational diabetes, multiple pregnancy, fetal anomaly, previous perinatal death, or any medical condition affecting pregnancy. Women were recruited and randomized in the early pregnancy and had vaginal microbial sampling performed at 16 weeks gestation prior to commencing assigned study intervention agent. The study was conducted at the National Maternity Hospital and UCD Perinatal Research Centre in Dublin between September 2016 and July 2019.

### Vaginal microbiota sampling, and metagenomic and functional signature data analysis

All women who had high vaginal swabs collected at randomization to the trial were included in this analysis. There was no difference in maternal meta-data or dietary composition between trial participants who had vaginal sampling and those who did not. Vaginal microbial swabs were taken at 16 weeks’ gestation from the posterior vaginal fornix after direct visualization of the cervix during speculum examination. Vaginal samples were immediately stored at −80°C and transferred to Teagasc Food Research Centre, Moorepark for analysis with shotgun metagenomic sequencing as previously described (40, 41). Compositional profiling of each samples was carried out with HUMAnN3 (v3.0) and the MetaPhlAn3 database (v30). Beta (between-sample) diversity was calculated using Bray-Curtis dissimilarity. Samples were visualized with the *ComplexHeatmaps* package (42) to display the relative abundance of each species. Community state types were assigned graphically using Heatmap, based on previously reported definitions (40, 43): community state type I (*Lactobacillus crispatus* dominant), community state type II (*Lactobacillus gasseri* dominant), community state type III (*Lactobacillus iners* dominant), community state type IV (low *Lactobacilli*), community state type V (*Lactobacillus jensenii* dominant). Alpha (within-sample) diversity was calculated using the Shannon’s index with the “diversity” function on the vegan package in R. Functional profiling was carried out with HUMAnN3 (v 3.0). Gene family output was normalized as copies per million reads and grouped according to gene ontology terms.

### Secretor and Lewis status detection

Participants who were breastfeeding provided breast milk samples using hand expression into a sterile container. Secretor and Lewis status were determined using methodology previously reported (41). Briefly, each breast milk sample was bathed with 100 µL of water, and the lipid was removed via centrifugation at 4,000 × *g* at 4°C. The aqueous layer was recovered and filtered through a 1-µm glass fiber plate. Proteins were ethanol precipitated and centrifuged; the upper liquid fraction was collected and dried. All samples were reconstituted in 100 µL of water and subjected to a sequential solid phase C18 and carbograph microplate extraction protocol. The human milk oligosaccharide (HMO) eluent was labeled with 2 -aminobenzamide by reductive amination (42), and free label and salts were removed using Diol plates. The separation of 2-AB-derivatized HMOs was carried out by ultra-high-performance liquid chromatography (UPLC) with fluorescence detection on a Waters ACQUITY UPLC H-class. The hydrophilic interaction liquid chromatography (HILIC) separation was performed using a Waters Ethylene Bridged Hybrid (BEH) Glycan column. The separation was performed using a linear gradient of 88%–43% MeCN at 0.56 mL/min over 35 min. An injection volume of 20 µL prepared in 88% (vol/vol) MeCN was used throughout. The system was calibrated using an external standard of 2-AB-labeled glucose oligomers to create a dextran ladder. After lipid extraction, all samples were processed on a 96-well plate using an eight-channel multipipette. Each sample was processed across three plates. The protocol was validated using commercially available HMO standards, and the observed glucose unit values obtained were comparable to those referenced in the literature. Fifty reproducible glycan peaks were resolved in all samples and integrated. The constituent HMO peaks were assigned. Secretor status was determined by the presence or near absence of 2′-fucosyllactose (2′-FL) and lacto-N-fucopentaose I (LNFP I), and Lewis status was assigned on the relative abundances of 3′-FL, lactodifucotetraose, LNFP II, LNFP III, and lactodifucohexaose-1.

### Environmental data collection

Environmental data in this study included the following data: demographics, socioeconomic factors, dietary pattern, exercise, and well-being data. Baseline environmental information was collected at recruitment, which included patient demographics and socioeconomic factors, such as maternal age, weight and BMI, ethnicity, education, and deprivation index. Smoking history was defined as either previous or current smoking. Parity and previous vaginal delivery data were collected. Ethnicity was self-reported as Caucasian (Irish and non-Irish European ancestry) or non-Caucasian (Asian, African, or Latino ancestry). Educated status was categorized as ceasing prior to completion of third level or completing third-level education (university-level education). Pobal Haase-Pratschke (HP Pobal) deprivation index was used to assess the economic advantage based on the participants’ home address (44). Medications, including antibiotics, were self-reported by participants. Participants completed a lifestyle survey to capture exercise pattern and well-being. The International Physical Activity Questionnaire was used to record exercise (45), and metabolic equivalent of tasks was calculated based on strenuousness, duration, and frequency of exercise activity (46). Participant well-being was assessed using the WHO-5 wellness tool (47) and validated for assessment of well-being with low scores being indicative of risk of depression (48).

All participants completed a 3-day food diary at recruitment. The diaries included type, amount, branding, and quantity of food consumed using either food packaging information or using common household measurements. Diet content was analyzed by nutritional composition using Nutritics Research Edition V.7 (49) to calculate daily intake of each macronutrient and micronutrient for each participant as well as daily intake of amino acids. Glycemic load and glycemic index were also calculated using Nutritics software. Glycemic load is a measure of impact of carbohydrates consumption on blood sugar (glucose) levels (50) and is calculated based on carbohydrate intake. Thus, glycemic load is a surrogate marker on the effect of carbohydrate intake on serum glucose and will heretofore be described with other macronutrients.

### Statistical analysis

Exploratory ordination and clustering of samples were performed using non-metric distancing scaling (NMDS) based on Bray-Curtis dissimilarity. Relationship between sample meta-data (demography, obstetric history, dietary data, exercise pattern, and well-being scores) and taxonomy was examined with vegan’s “envfit” function. The envfit function performs multiple regression of the meta-data covariates against the NMDS ordination axes to generate a *P* value by permutation. Covariates with high collinearity were excluded (Fig. S2). Explanatory ordination with redundancy analysis was then performed. Functional signatures, including biological processes (BP), cellular component (CC), and metabolic functions (MF), were then examined against meta-data and vaginal community state type using similar ordination approach incorporating all individual functional gene pathway data. Maternal covariates were next examined against Shannon alpha diversity, using regression models for continuous variables, and Mann Whitney *U* comparisons to compare diversity between categorical outcomes. Regression models were adjusted for confounders with known associations with the vaginal microbiome, including maternal age, weight, parity, ethnicity, education status, and deprivation index. Covariates were then compared between community state type groups, excluding groups with cohorts less than 10. Categorical covariates were compared using Fisher’s exact test. Continuous data were assessed for normality using mean, median, skewness kurtosis, and Shapiro-Wilk test. For parametric continuous data, ANOVA testing was performed to compare means between each CST group. For non-parametric continuous data, Kruskal Wallis testing compared medians between groups between CST group. Finally, subgroup analysis was also conducted according to secretor status. All data were adjusted for multiple comparisons using Benjamini-Hochberg or Bonferroni procedures. Statistical analysis was performed using R Statistical Software (version 4.2.2, 31 October 2022). Graphics and envfit analysis were created using packages, including utilizing “tidyverse,” “ggplot2,” and “vegan” packages (51–53).

### Role of the funding source

Science Foundation Ireland, who provided the funding, and the industry partner, who provided the probiotic and placebo, had no role in the design of the study and did not have access to study data or analysis of data.

## RESULTS

**Fig 1:**
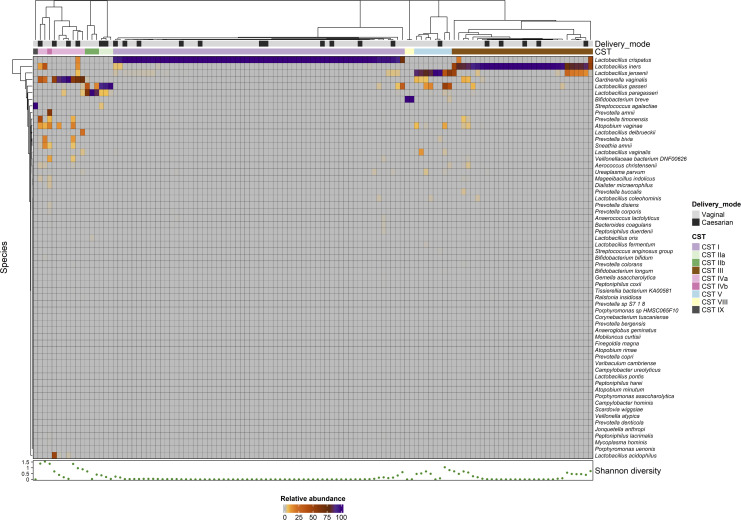
Vaginal microbial composition at 16 weeks’ gestation in a healthy pregnancy cohort. Heatmap of vaginal microbial composition. Graphical representation of the dominant species in each vaginal sample shown on heatmap to form nine distinct vaginal community state type groups based on dominant species.

A total of 119 women with uncomplicated pregnancies had vaginal samples collected at 16 weeks of pregnancy, and 118 completed dietary assessment. Following metagenomic sequencing, an average of 565,192 high-quality paired-end reads was obtained per sample (median 350,006). All samples were graphically represented by heatmap analysis (Fig. 1). The dominant species was *Lactobacillus crispatus*, with relative abundance over 95% in 51.3% (61/119) samples. Prevalence of low lactobacilli abundance was 8.4% (10/119). Each specimen was assigned to vaginal community state type based on dominant species. Two participants had *Bifidobacterium breve* abundance over 99% (assigned CST VIII), and one participant had 100% abundance of streptococcus agalactiae (assigned CST IX). Baseline population characteristics are included in Table 1.

**TABLE 1:** Baseline population characteristics of the study population in healthy uncomplicated pregnancy at 16 weeks’ gestation

Characteristic		*N* (%) or mean ± SD
Maternal age	In years (mean, SD)	33.3 ± 3.9
Parity	Nulliparous	66 (55.9)
	Multiparous	52 (44.1)
	Previous vaginal delivery	40 (33.9)
	Previous cesarean delivery only	9 (7.6)
Previous breastfeeding	After prior pregnancy	40 (33.9)
Body mass index	Units (mean, SD)	25.0 ± 3.2
Ethnicity	Caucasian (European descent)	103 (92.8)
	Non-Caucasian (Asian, Latino, and African descent)	8 (7.2)
Education status	Completed third level	102 (87.9)
Smoking	Current or previous history	46 (39.0)
METS (minutes)	Per week (mean, SD)	1154.8 ± 1617.4
WHO-5 well-being score	Percentage (mean, SD)	61.9 ± 21.2
Antibiotic use	In early pregnancy	10 (8.7)
Community state type	CST I	63 (53.4)
	CST II	6 (5.1)
	CST III	28 (23.7)
	CST IV	10 (8.4)
	CST V	8 (6.8)
	CST III	2 (1.7)
	CST IX	1 (0.8)
Secretor status (*n* = 72)	Positive	54 (75.0)
	Negative	18 (25.0)

### Maternal meta-data and vaginal taxonomy in early pregnancy

**Fig 2:**
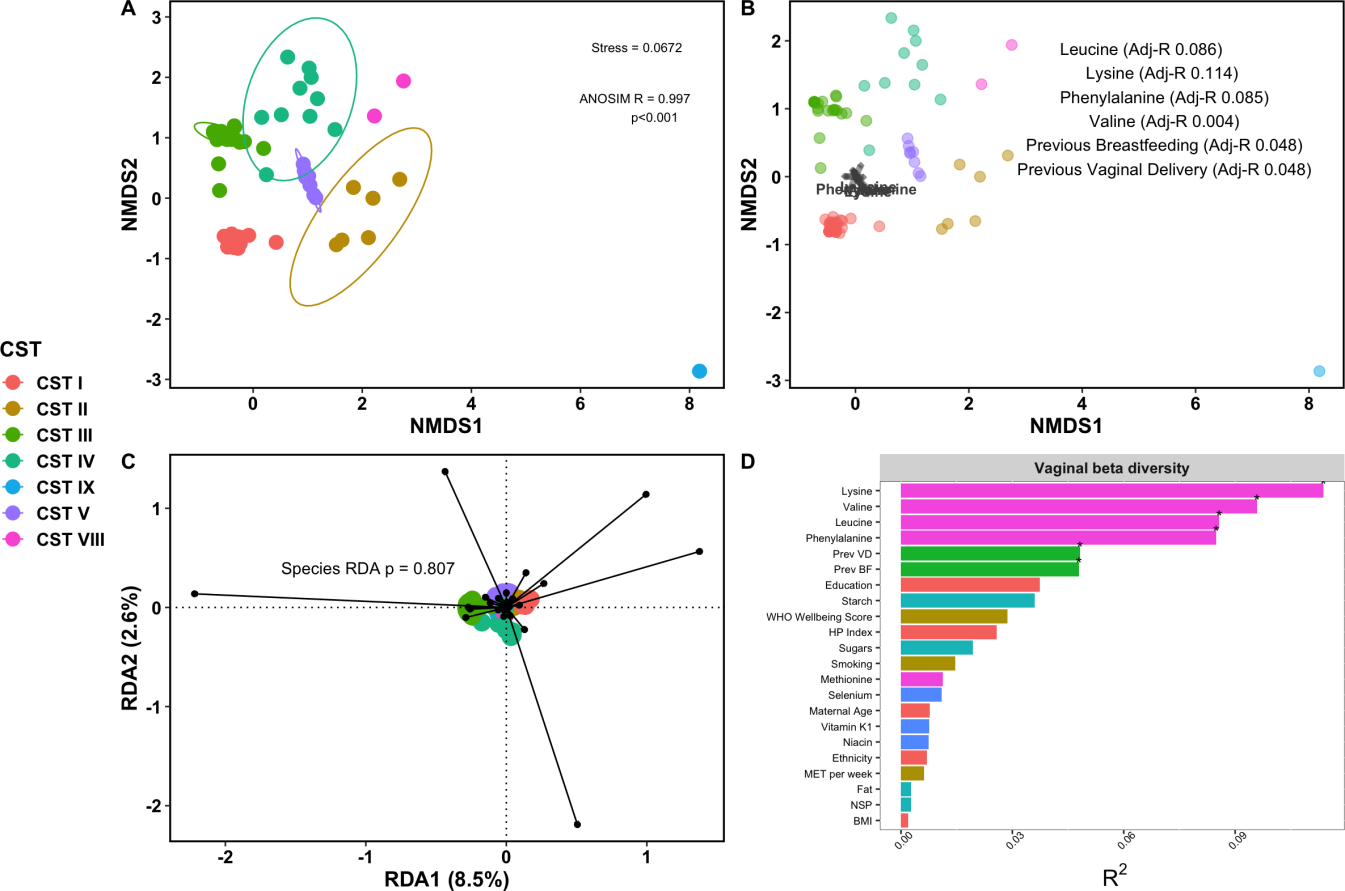
Vaginal species composition at 16 weeks’ gestation in healthy pregnancy cohort. (A) NMDS ordination plot of vaginal microbial species using non-metric multidimensional scaling based on Bray-Curtis dissimilarity. (A) is annotated with the stress of the model (amount of variability unexplained by the NMDS ordination). (B) Envfit model plotted on functional ordination, displaying variables with significant (*P* < 0.05) association with species plot, and adjusted-*R* values of significant covariates annotated. (C) Redundancy analysis of maternal meta-data against vaginal species composition, showing variation in the data attributable specifically to covariates identified in envfit model on the *x*-axis. (D) Envfit barchart of covariate adjusted *R* squared and *P* value when comparing difference in centroids of each covariate relative to the total variation, with significant covariates denoted with asterisk.

Ordination of vaginal species data is shown in Fig. 2A (ANOSIM-R = 0.997, *P* < 0.001, Fig. S1). Covariates with strongest association with vaginal beta diversity included dietary lysine (adj-*R*^2^ 0.113, *P* = 0.002), valine (adj-*R*^2^ 0.096, *P* = 0.004), leucine (adj-*R*^2^ 0.086, *P* = 0.003), and phenylalanine (adj-*R*^2^ 0.085, *P* = 0.005, Fig. 2D). Among demographic and obstetric covariates, the only factors with significant differences in beta diversity were previous vaginal delivery (adjusted *R*^2^ 0.048, *P* = 0.003) or history of breastfeeding (adj-*R*^2^ 0.045, *P* = 0.004). There was no relationship between beta diversity and demographic or lifestyle factors, including ethnicity, education, maternal well-being, or exercise patterns (Fig. 2D). Redundancy analysis showed these 22 maternal factors accounted for 16.7% variation in vaginal taxonomy (constrained proportion 0.1674, Fig. 2C).

### Maternal meta-data and functional signature of the vaginal microbiome in early pregnancy

**Fig 3:**
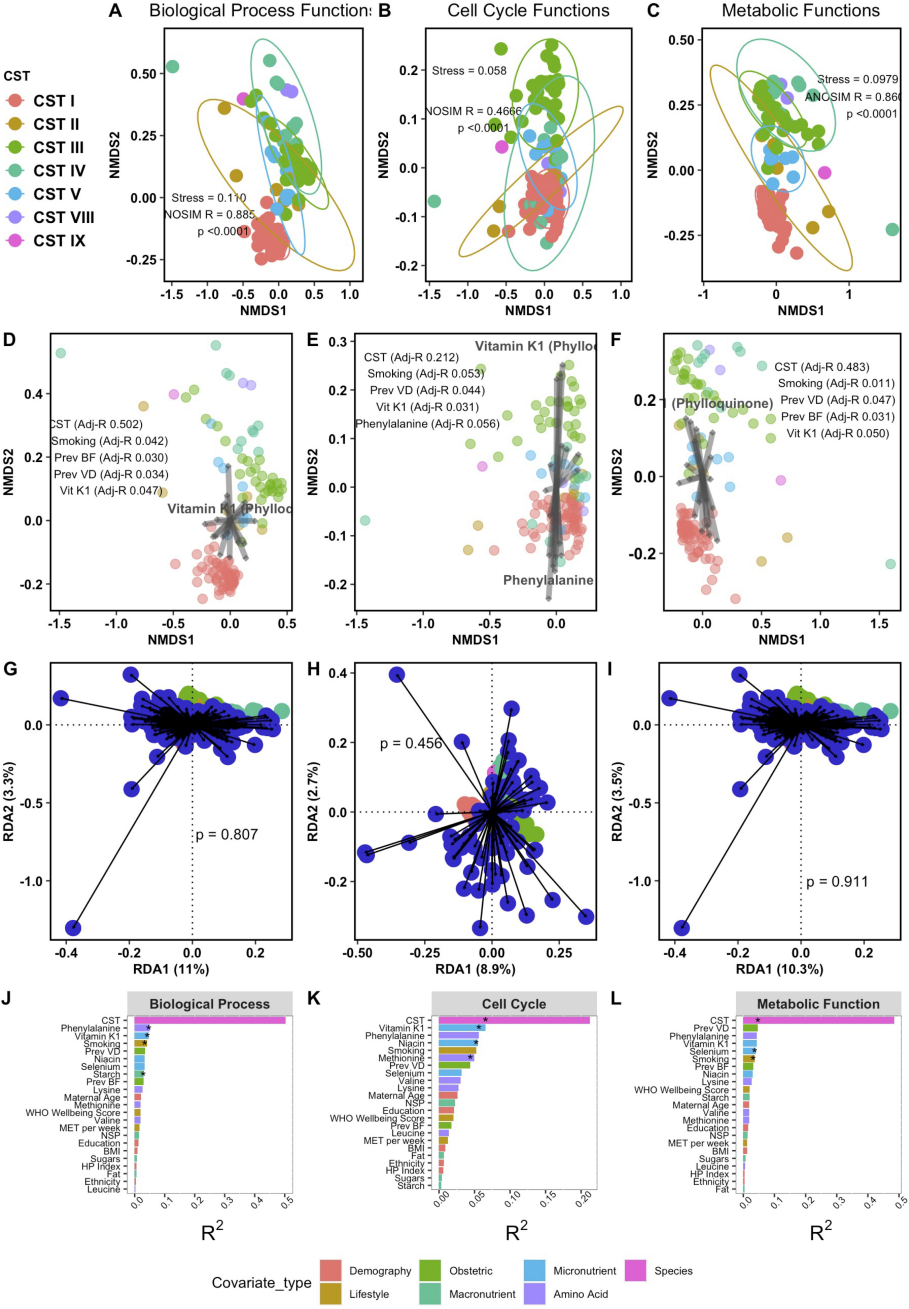
Analysis of functional gene ontology of the vaginal microbiome at 16 weeks’ gestation. (**A–C**) NMDS ordination of functional data sets BP, CC, and MF annotated with the stress of the model (amount of variability unexplained by the NMDS ordination). (**D–F**) Envfit covariates plotted on functional ordination plots, displaying variables with significant (*P* < 0.05) association with species plot, and adjusted-*R* values of significant covariates annotated. (**G–I**) Redundancy analysis of meta-data against function, showing variation in the data attributable specifically to covariates identified in envfit model on the x-axis. (**J–L**) Envfit barchart of covariate adjusted *R* squared and *P* value when comparing difference in centroids of each covariate relative to the total variation within the function, with significant covariates denoted with asterisk. CC, cell cycle functions; RDA, redundancy analysis; VD, vaginal delivery; BF, breastfeeding; NSP, non-starch polysaccharides; MET, metabolic equivalent of tasks.

Ordination of functional pathways of vaginal microbiome, including biological processes (Fig. 3A, ANOSIM R 0.885, *P* < 0.001), cellular components (Fig. 3B, ANOSIM-R 0.4655, *P* < 0.001), and metabolic functions (Fig. 3C, ANOSIM 0.860, *P* < 0.001), is described. Interaction of these pathways with maternal covariates (Fig. 2D through F and Fig. 2J through L) showed the single greatest factor associated with all three functions was vaginal composition, represented by vaginal community state type (biological processes and CST adj-*R*^2^ 0.502, *P* < 0.001; cellular components and CST adj-*R*^2^ 0.086, *P* = 0.003; metabolic functions and CST adj-*R*^2^ 0.086, *P* = 0.003). Biological processes also differed with maternal smoking (adj-*R*^2^ 0.042, *P* = 0.010), previous breastfeeding (adj-*R*^2^ 0.029, *P* = 0.030), prior vaginal delivery (adj-*R*^2^ 0.034, *P* = 0.017), and vitamin K1 (adj-*R*^2^ 0.047, *P* = 0.044). Cellular component function was associated with maternal smoking (adj-*R*^2^ 0.037, *P* = 0.011), previous breastfeeding (adj-*R*^2^ 0.032, *P* = 0.027), prior vaginal delivery (adj-*R*^2^ 0.047, *P* = 0.005), vitamin K1 (adj-*R*^2^ 0.037, *P* = 0.011), and phenylalanine (adj-*R*^2^ 0.056, *P* = 0.041). Metabolic function was associated with maternal smoking (adj-*R*^2^ 0.053, *P* = 0.010), prior vaginal delivery (adj-*R*^2^ 0.044, *P* = 0.011), and vitamin K1 (adj-*R*^2^ 0.043, *P* = 0.050). Redundancy analysis showed maternal meta-data accounted for 8.9%–11% of variation in microbiota functional signals (Fig. 3G through I, BP 11%, *P* = 0.807; CC 8.9%, *P* = 0.456, MF 10.3%, *P* 0.911).

### Maternal obstetric factors and vaginal composition

**Fig 4:**
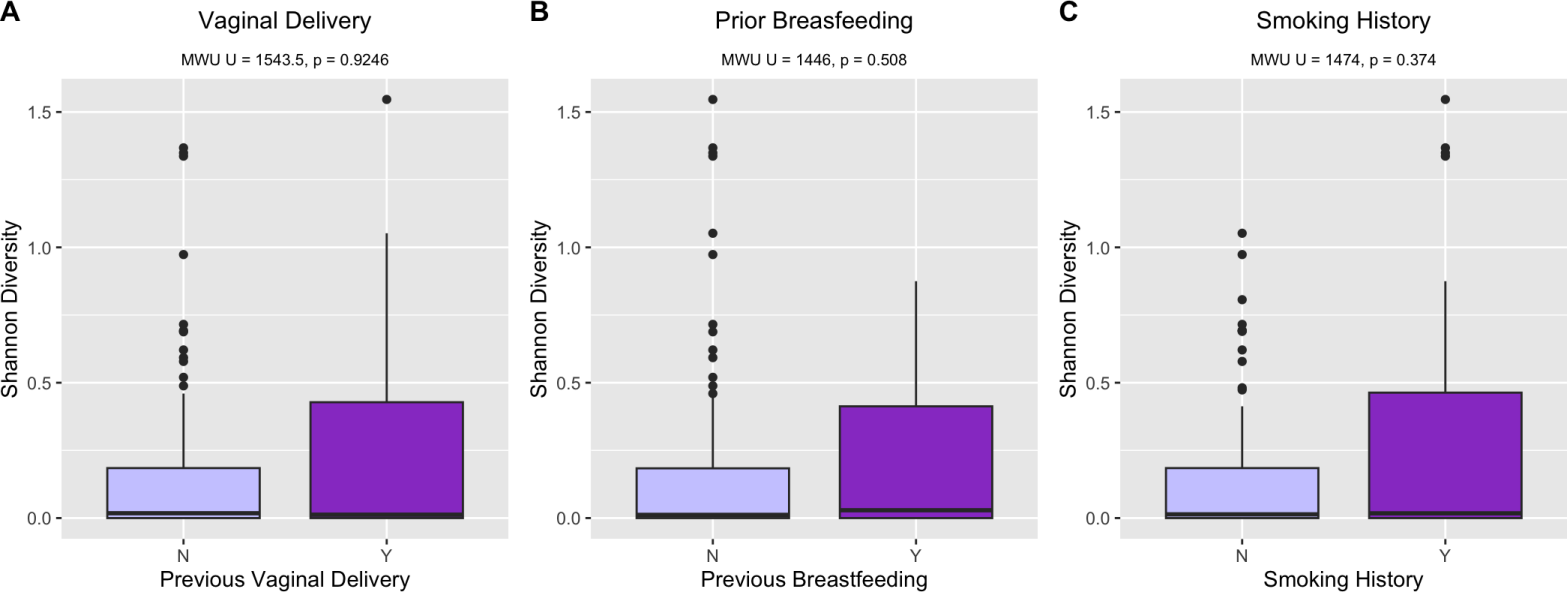
Previous vaginal delivery (A), breastfeeding history (B), and maternal smoking status (C) and vaginal Shannon alpha diversity in early pregnancy.

There was no significant correlation alpha diversity and previous vaginal delivery (Fig. 4A, Mann-Whitney U test (MWU) = 1554.5, *P* = 0.925), history of breastfeeding (Fig. 4B, MWU = 1446.0, *P* = 0.508), or maternal smoking (Fig. 4C, MWU 1474.0, *P* = 0.374). Parity and history of breastfeeding had a significant association with assignment of CST. Increased prevalence of CST III or IV compared to CST I was observed with multiparity (60.0%–62.1% vs 23.8%, *P* < 0.001) history of previous vaginal delivery (40.0%–51.7% vs 17.5%, *P* = 0.003) and history of breastfeeding (40.0%–50.0% vs 18.0% *P* = 0.005, Table S1).

### Maternal dietary factors and vaginal alpha diversity

**Fig 5:**
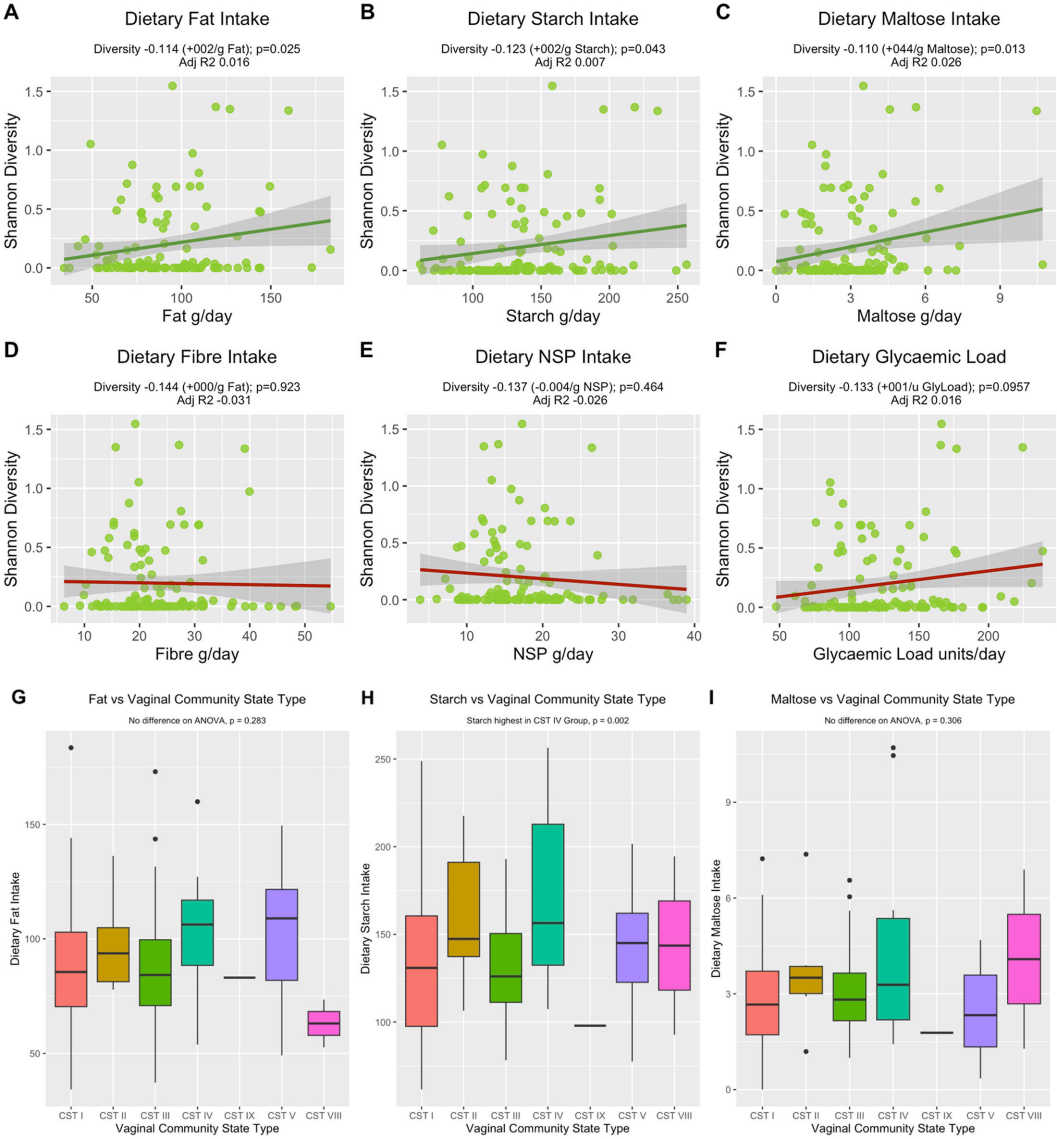
Macronutrients daily nutritional intake and (**A–F**) vaginal Shannon alpha diversity and (**G–I**) vaginal community state type in early pregnancy. All regression models are adjusted for maternal confounders, including maternal age, weight, parity, ethnicity, education status, and deprivation index.

Maternal factors were examined against alpha “Shannon” diversity of the vaginal samples. Dietary fat, starch, and maltose were all positively correlated with alpha diversity (Fig. 5A through C; fat +0.002 SD/g, *P* = 0.025; starch +0.002 SD/g, *P* = 0.043; maltose +0.44 SD/g, p=0.013). Daily starch consumption was also highest in community state type IV (*P* = 0.002). ANOVA and post hoc testing of dietary intake and CST showed that CST IV, with highest Shannon diversity, had significantly higher intakes of carbohydrates and starch and higher dietary glycemic load compared to CST I and III (Table S1; Fig. S5). Secretor status was established in 72 of the 119 participants, compared to participants who did not have secretor status assessed; those with known secretor status had higher HP index (8.82 ± 8.82 vs 4.35 ± 10.02), lower BMI (24.54 ± 2.81 vs 25.53 ± 3.65) and higher dietary energy (2202.71 ± 454.77 kCal/day), protein (91.65 ± 20.66 vs 89.00 ± 20.61 g/day), fat (96.60 ± 28.03 vs 84.34 ± 23.69 g/day), carbohydrate (241.25 ± 58.51 vs 216.73 ± 216.73 ± 55.23 g/day), NSP (18.59 ± 6.47 vs 16.49 ± 6.58 g/day), and glycemic load (130.19 ± 35.87 vs 116.89 ± 33.96 units/day). Secretor-positive status was associated with stronger positive correlations between macronutrient intake and alpha diversity (fat +0.003 SD/g, *P* = 0.050; starch +0.002 SD/g, *P* = 0.086; maltose +0.033/ g, *P* = 0.137). By comparison, this same positive correlation was not observed in secretor-negative women (fat −0.000 SD/g, *P* = 0.766; starch −0.005 SD/g, *P* = 0.038; maltose −0.003 SD/g, *P* = 0.639). Lewis status was determined for the 72 samples, with 69 positive and 3 negative, with this small cohort size limiting full analysis by Lewis status. No correlation was observed between alpha diversity and dietary amino acids or micronutrients (Fig. S3 and S4) or CST (Table S1).

## DISCUSSION

This study reports that several environmental factors are associated with the vaginal microbiome in early pregnancy. Major factors shown to be associated with vaginal microbial composition included a previous vaginal birth, prior breastfeeding, glycemic load, and dietary intake of starch and SCFA precursor amino acids. Several recent studies have linked parity to vaginal microbial composition (12, 54). Mode of birth is detailed in several studies, including Kervinen et al. who found differences in relative abundance of *L. crispatus and L. gasseri* were only observed after vaginal delivery or birth by emergency cesarean (12) and Costello et al. found vaginal birth was followed by significant increases in Shannon alpha diversity when compared to cesarean section (55). Both of these are in-line with our findings that previous vaginal delivery alters vaginal taxonomy and is associated with reduced prevalence of CST I compared to women with a prior cesarean section. The dysbiotic effect of vaginal birth is mostly marked within the first year postpartum (11, 56–58). While multiparity itself is not known to increase risk of preterm birth, both short interpregnancy interval and grand multiparity are known risk factors for preterm birth (59, 60). Vaginal microbiota dysbiosis in these scenarios offers a possible explanation for increased risk of subsequent preterm birth, but this requires further population-specific examination. Analysis from this study cohort recently showed the transfer of microbiota to the newborn has been linked with sharing events, including spontaneous onset of labor, spontaneous rupture of membranes, and vaginal delivery, affecting neonatal gut health (41). To complement this, here, we observe long-reaching association between vaginal delivery and vaginal microbial composition. The authors hypothesize that vaginal delivery may exert local structural mucosal changes in the vagina and increase mucosal permeability to bacteria. Given changes in gut microbiota observed postdelivery, another potential pathway is via exert immune or mucosal effects in the gut which may drive this postnatal vaginal dysbiotic picture (41, 56).

The association between breastfeeding, vaginal species, and assignment to CST III or IV has been reported in animal studies (61), but there has been limited documentation of this in human populations. We hypothesize this may relate to the relative hypo-estrogenic state induced by breastfeeding. In pregnancy, placenta production of estrogen is thought to drive vaginal epithelial maturation and accumulation of glycogen, a polysaccharide that supports *Lactobacillus* spp. colonization and promotes *Lactobacillu*s spp. dominance (62). The postnatal depletion of estrogen has previously been linked to increased diversity and dysbiosis (11, 63), and it may be that breastfeeding prolongs this hypo-estrogenic *Lactobacillus* spp. deplete state. There may also be links with vaginal birth, as the majority of the multiparous group in this cohort had delivered vaginally, with small comparative group who had never breastfed. Further research is required to examine these factors and the relationships in the context of postnatal temporal hormonal fluctuations.

Intriguingly, AA, the precursors of short-chain fatty acids, have the strongest dietary associations with vaginal microbial composition in early pregnancy. There was no correlation between these AA and alpha diversity or assignment to CST, and the exact nature of their interaction is yet to be elucidated. Although there have been some studies linking biogenic amino acid levels in the vaginal fluid to bacterial vaginosis (BV) (64, 65), associations between dietary intake of SCFA precursor amino acids and the vaginal microbial species have not yet been described. The dietary-vaginal axis interplay may be explained through the gut as the intermediate regulatory player. Dietary amino acids have dual function at the gut-microbial interface, where they are precursors for the SCFAs during microbial-driven fermentation, and they support growth and survival of microbiota and assist in energy and protein homeostasis of gut bacteria (66). Linking intake of dietary amino acids to vaginal microbial composition needs further investigation to understand the mechanisms that underlie this association.

Dietary macronutrient profiles are significantly associated with vaginal microbiome in early pregnancy. Fat, starch, and maltose are significantly associated with vaginal alpha diversity, particularly in secretor-positive women, and higher consumption of carbohydrates, starch, and glycemic load is all linked with community state type IV. These echo studies previously linked these macronutrients to dysbiotic conditions such as bacterial vaginosis (67). Despite the evidence around fiber and non-starch polysaccharides and gut microbial health, there appeared to be no association with the vaginal microbiome, in keeping with other smaller studies (68). Where optimal pregnancy health is seen with low alpha diversity, there is potential for dietary interventions, namely optimized macronutrient consumption, to reduce diversity and associated adverse outcomes. These dietary findings may be explained by further examination of the interplay between gut and vagina microbiota and metabolomes, but this interplay is yet to be fully understood.

The functional signature of the vaginal microbiome in healthy pregnancy appears to be mainly associated with vaginal species composition. Community state type was the strongest associative factor with all functional pathways, when compared against maternal demographic lifestyle and dietary factors. Maternal smoking and consumption of some nutrients such as vitamin K1 and phenylalanine also play a role but to a lesser degree than vaginal taxonomy. Smoking is known to alter the vaginal metabolome profile (69), vaginal epithelial health, and associations with adverse vaginal conditions, such as bacterial vaginosis and persistent human papillomavirus (69, 70), which fit our study’s observation of its link with functional signature of the vaginal microbiome in pregnancy. Both vitamin K and phenylalanine are players in gut microbial function and health. Vitamin K has been observed to improve gut dysbiosis and intestinal inflammation, with subsequent benefits at distant host sites, such as the brain and central nervous system (71). Phenylalanine, a dietary precursor to tyrosine, is metabolized by gut microbiota to produce Short-chain fatty acids. The authors hypothesize that its relationship with vaginal microbial function may be through systemic immune regulation to alter vaginal species or by influencing metabolism of vaginal microbiota directly. Although these factors or maternal smoking, dietary vitamin K, and phenylalanine have a smaller role, we illustrate the interaction between host and microbial function occurs mainly through species composition. These findings are helpful when interpreting the interplay between diet, species, and functional signature of the vaginal niche in early pregnancy.

Although plausible to be associated with the vaginal microbiome, given their link with other microbial niches (72), exercise and well-being did not feature in this pregnancy cohort as a significant covariate with no relationship with vaginal microbial species, alpha diversity, and assignment to CST. There are limited data on the relationship between exercise and the vaginal microbiome, with just one small study of 26 women previously identifying an association between high intensity exercise and increased alpha diversity (73). Exercise is an integral part of human health and has numerous health benefits, particularly in pregnancy (74–76). Further examination in larger scale studies will be helpful to establish if there are benefits of exercise directly on the vaginal microbiome or even indirectly through its action on other microbial niches. Well-being and mental health are also known to be directly associated with the gut microbiome through the gut-brain axis (77), and previous studies have noted correlation between stress scores and vaginal dysbiosis (78, 79). The relationship between brain function and gut and vaginal microbiota, particularly in the context of pregnancy, also warrants further exploration.

### Strengths and limitations

Strengths of this study include the expansive profile of environmental exposures captured along with the examination of these factors’ interaction with one another and the vaginal microbiome. Data processing with community ecological software (51) also enabled exploratory regression analysis with numerous covariates in order to identify significant factors. The robust nature of sample collection minimized pre-analytical variability. High-quality microbial processing with shotgun metagenomic sequencing allowed a degree of analysis that captured species strains such as *Bifidobacterium* that can be challenging to sequence with traditional metagenomic approaches. The functional signature of the vaginal microbiome is also captured to illustrate the complex interactions between the host and microbe, which is limited in the setting of pregnancy. Selection bias is acknowledged, as research participants were healthy, well-educated women with a BMI under 35, without medical comorbidities and tended to have low ethnical diversity (*n* = 6 non-Caucasian participants). With a relatively small sample size of 119 participants, there may be additional significant environmental factors. It will be critical to re-examine interactions between dietary exposures and vaginal microbiome in population-based settings such as the SweMami project (80) in larger cohorts. However, despite the cohort size, there were a number of significant novel findings around dietary amino acid consumption and links with the vaginal microbial species, and this will be helpful in guiding interpretation and analysis in future larger observational studies.

### Implications

Environmental factors play an integral role in physical and mental health, physiology, and immune health but may also enable pathogenic processes to cause disease. As we eat, move, and think each day, all of these factors are thought to influence an individual’s microbial ecosystem, both directly on the vaginal microbiota and indirectly through the regulatory effect of other niches, such as the gastrointestinal tract on the phenotypic response in the vaginal microbiota. It is obvious that understanding the interplay between the vaginal microbiota and environmental factors is critical. The novel findings reported here highlight that the most significant of these are previous vaginal delivery and dietary exposure of amino acids. The dietary associations reported here add to the conversation around the gut-vaginal microbial axis and require further investigation to establish how the association of dietary amino acid consumption is exerted on vaginal microbial composition.

There are a small number of interventional trials examining precision dietary interventions to improve the microbial health of non-vaginal niches in non-pregnant populations. One trial showed habitual diet interventions, such as Mediterranean diet, can improve gut microbial health, reduce inflammatory profile, and promote healthier aging (81). Another recent study showed high fiber polyphenol-enriched and vegetable protein-based diet provides benefits for the composition of fecal microbiota and may offer potential therapies for the improvement of glycemic control, dyslipidemia, and inflammation (82). However, before environmental-precise interventions are undertaken to optimize microbial health in pregnancy, a deeper understanding is required on dietary pattern and vaginal microbial health parameters, both before and during pregnancy. Large population-based studies such as the SweMaMi project (80) will hopefully be able to further illuminate the complex and integral relationship between the dietary-gut-vaginal axis.

### Conclusion

This study reveals that vaginal taxonomy is associated with previous vaginal birth, history of breastfeeding, dietary consumption of amino acids, and an individual’s macronutrient profile. Amino acids have the most significant association with vaginal taxonomy in early pregnancy. Dietary fat and starch are linked with increased alpha diversity, especially in secretor-positive women, and increased carbohydrates, starch, and glycemic load are associated with community state type IV. Functional signature of the vaginal microbiome differs most strongly with vaginal taxonomy, but maternal smoking, vitamin K, and phenylamine also play a role. These data provide an intriguing and novel insight into the complex interactions between the diet, human gut, and vaginal microbiota composition and function.

## Data Availability

Data requests can be sent to the corresponding author. The full MicrobeMom clinical trial protocol is available on ISRCTN website (https://www.isrctn.com/ISRCTN53023014?q=&filters=&sort=&offset=98&totalResults=15003&page=1&pageSize=100&searchType=basic-search). The primary outcome paper is available at https://www.ajogmfm.org/article/S2589-9333(23)00136-2/fulltext, and detailed mapping of the maternal gut microbial composition is detailed at https://www.nature.com/articles/s41467-023-38694-0. All raw sequencing data used in this study are available in the ENA repository under accession number PRJEB48251. Script used in the analysis is available at: https://github.com/gilliancorbett/Maternal-Factors-Vaginal-Microbiome---MicrobeMom-RCT-Secondary-Analysis.

## SUPPLEMENTAL MATERIAL

Supplemental figures and table spectrum.01130-24-s0001.docx Fig. S1 to S5; Table S1.

ASM does not own the copyrights to Supplemental Material that may be linked to, or accessed through, an article. The authors have granted ASM a non-exclusive, world-wide license to publish the Supplemental Material files. Please contact the corresponding author directly for reuse.
