# Supplementary material for: Dietary amino acids, macronutrients, vaginal birth, and breastfeeding are associated with the vaginal microbiome in early pregnancy
Source: Microbiol Spectr. 2024 Oct 4;12(11):e01130-24. doi: 10.1128/spectrum.01130-24 (PMC11537119; doi:10.1128/spectrum.01130-24)
Supplement: Supplemental figures and table — Fig. S1 to S5; Table S1. [file spectrum.01130-24-s0001.docx]

**Supplementary Data.**

**Figure S1. ANOSIM Plots of Each NMDS Ordination comparing the mean of ranked dissimilarities between vaginal CST. All four models are statistically significant, demonstrating a statistically significant difference between microbial communities of each community state type.**

**
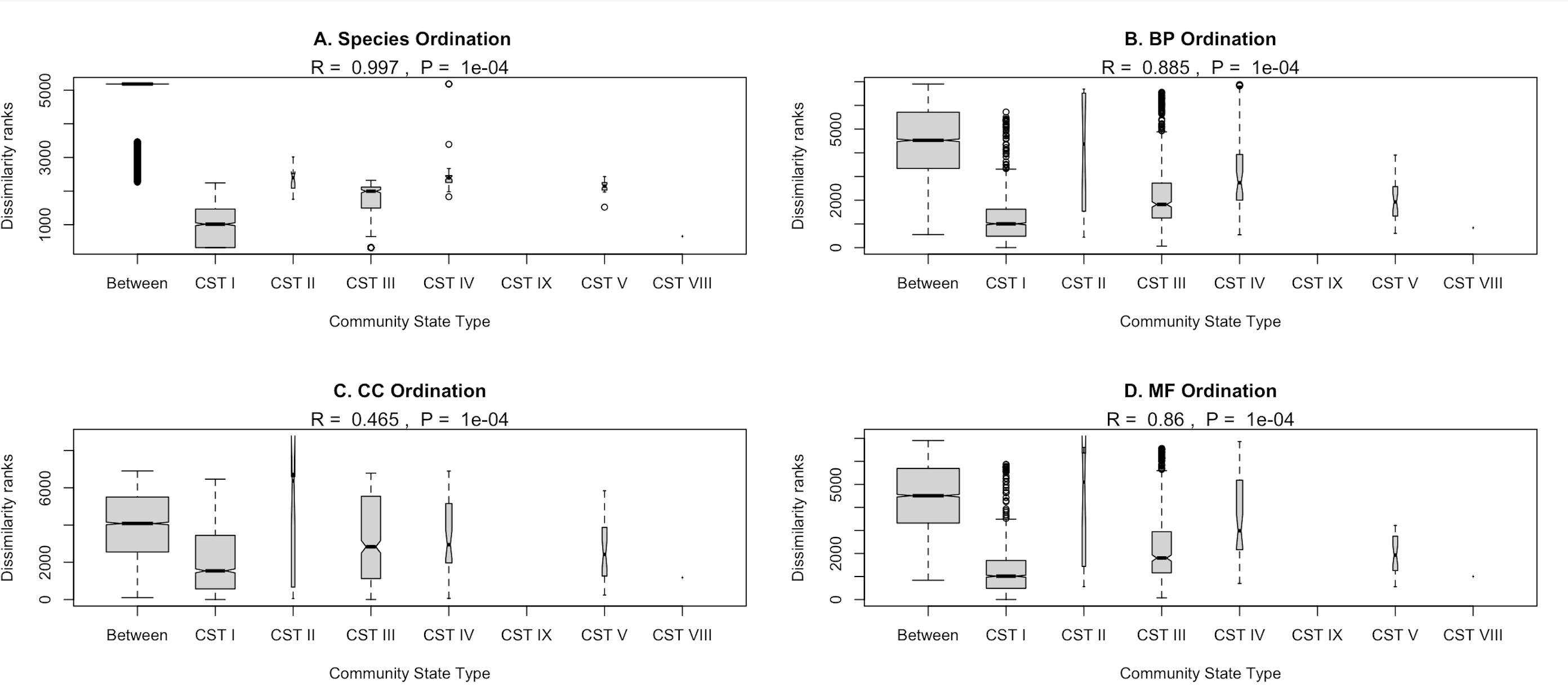
**

**Figure S2. Heatmap of environmental data collinearity**

**
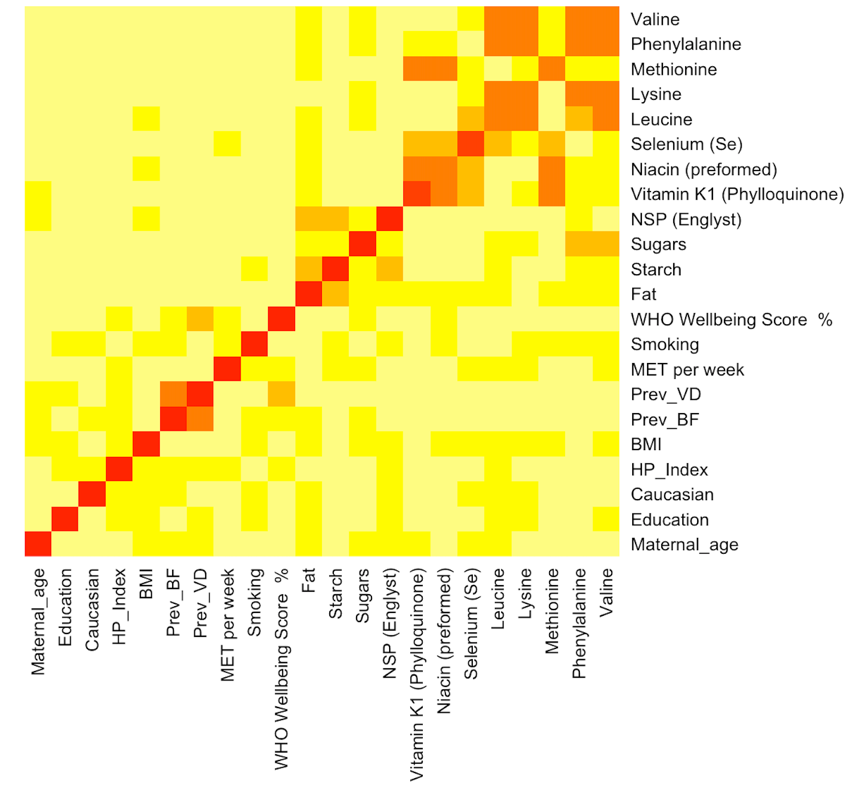
**

**Figure S3. Amino Acid Daily Nutritional Intake and Vaginal Shannon Alpha Diversity in Early Pregnancy.** No relationship between amino acid intake and diversity.


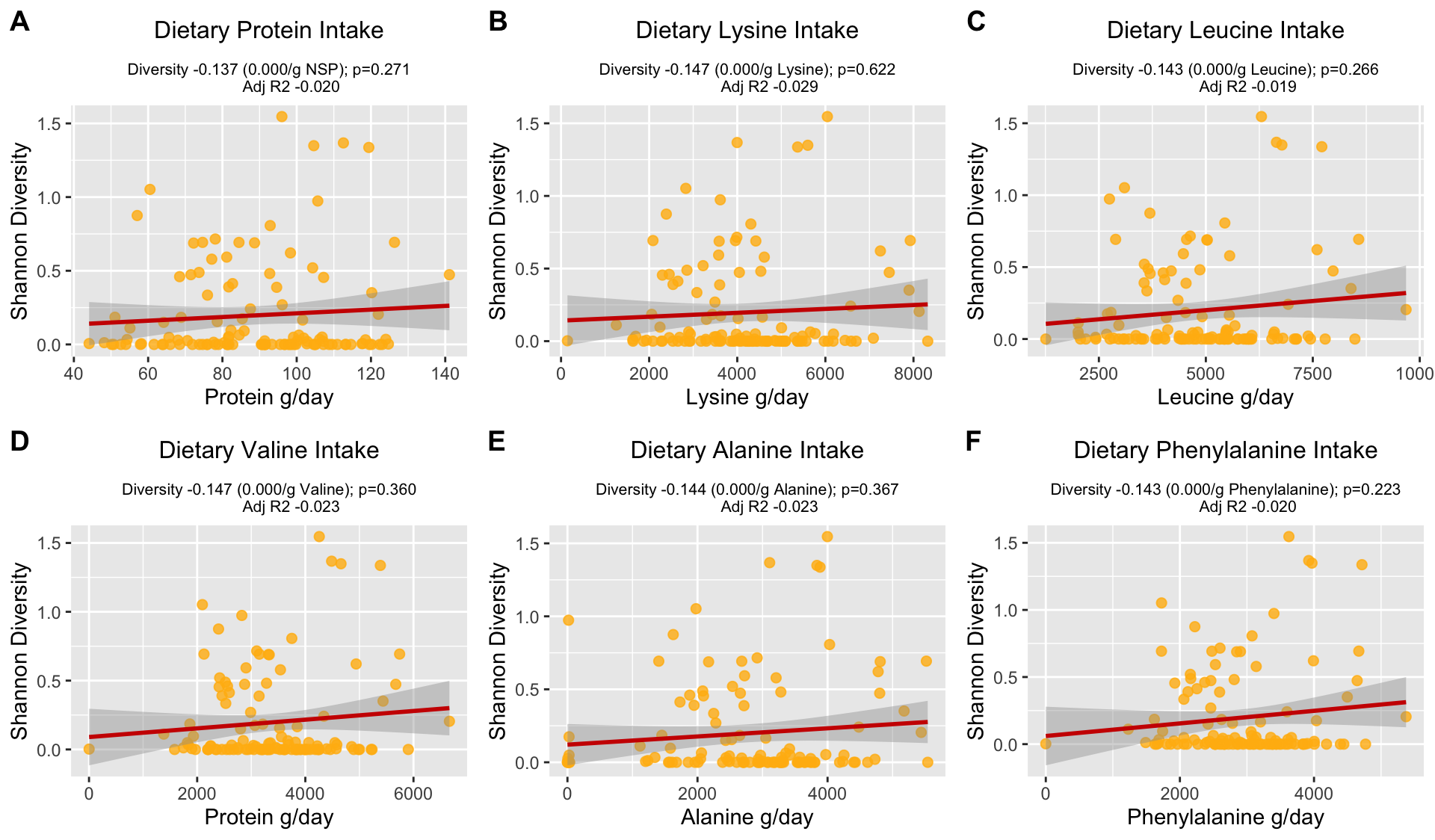


**Figure S4. Micronutrients Daily Nutritional Intake and Vaginal Shannon Alpha Diversity in Early Pregnancy.** No relationship between Niacin, Vit K1 or Selenium intake and diversity.


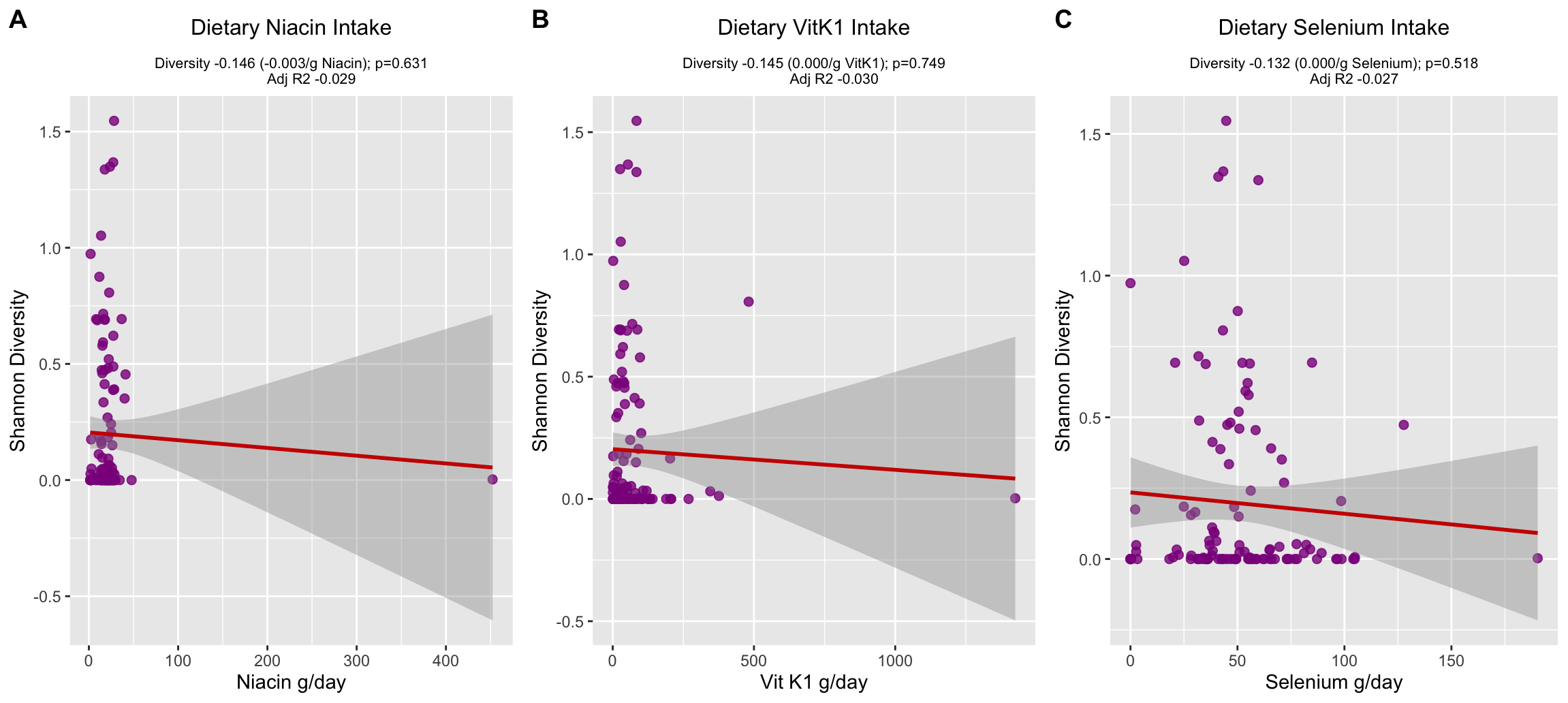


**Supplementary Table S1. This table shows results of comparative analysis of environmental factors between community state types, after adjustment for confounders.**

| **Characteristic** | **CST I (n=63)** | **CST III (n=27)** | **CST IV (n=10)** | **P Value** |
| --- | --- | --- | --- | --- |
| Nulliparous | 48 (76.2%) | 11 (37.9%) | 4 (40.0%) | **<0.001** |
| Multiparous | 15 (23.8%) | 18 (62.1%) | 6 (60.0%) |  |
| Previous vaginal delivery | 11 (17.5%) | 15 (51.7%) | 4 (40.0%) | **0.003** |
| Previous caesarean delivery only | 4 (6.6%) | 3 (10.0%) | 0 (0.0%) | 0.718 |
| Breastfeeding History | 11 (18.0%) | 15 (50.0% | 4 (40.0%) | **0.005** |
| WHO Well-being Score* | 68.0 (64.0-76.0) | 64.0 (48.0 – 76.0) | 64.0 (43.0-73.0) | 0.170 |
| Metabolic equivalent per week* | 420.0 (150.0-1650.0) | 792.5 (204.0-1655.0) | 495.0 (30.0-1858.5) | 0.677 |
| Protein | 90.3 (19.7) | 83.8 (22.2) | 91.5 (20.3) | 0.325 |
| Fats | 88.9 (27.1) | 88.6 (29.1) | 103.6 (29.5) | 0.283 |
| Carbohydrates (g/d) | 222.8 (52.3) | 219.7 (52.2) | 279.47 (78.6) | **0.002** |
| Starch (g/d) | 132.1 (41.2) | 129.8 (29.7) | 172.1 (51.0) | ***0.011*** |
| Maltose* | 2.7 (1.7-3.7) | 2.8 (2.2-3.9) | 3.3 (2.0-6.8) | 0.306 |
| Fibre | 23.3 (8.7) | 22.0 (9.3) | 26.8 (8.5) | 0.344 |
| Non-Starch Polysaccharides | 17.7 (7.1) | 15.8 (5.3) | 19.5 (7.2) | 0.245 |
| Sugars | 88.6 (30.0) | 86.6 (35.6) | 101.5 (33.0) | 0.434 |
| Glycaemic Load | 118.4 (32.5) | 118.4 (33.0) | 152.0 (47.8) | **0.006** |
| Glycaemic Index | 53.2 (6.2) | 53.3 (5.2) | 53.9 (4.0) | 0.937 |
| Lysine | 4284.0 (1348.0) | 3734.4 (1611.2) | 3907.1 (1356.3) | 0.068 |
| Leucine | 4923.1 (1536.2) | 4493.2 (1399.9) | 4784.6 (1973.1() | 0.197 |
| Valine | 3477.5 (909.2) | 3064.9 (2083.0) | 3431.4 (1202.1) | 0.057 |
| Arginine* | 3372.2 (2618.3-4044.5) | 2673.9 (1958.1-3663.0) | 3016.1 (1182.9 – 4224.3) | 0.114 |
| Alanine* | 4776.8 (2387.0 – 3593.9) | 2386.7 (1720.6 – 3262.3) | 2681.8 (1059.6 – 3848.1) | 0.135 |
| Glycine* | 2517.8 (1922.1 – 3045.0) | 2017.7 (1426.4 – 2710.5) | 2101.7 (822.1 – 3263.5) | 0.179 |
| Glutamate | 39.7(12.5) | 34.9 (13.7) | 34.2 (9.9) | 0.198 |
| Threonine | 2813.6 (754.8) | 2429.0 (1145.1) | 3032.9 (1213.6) | 0.105 |

Note: Vaginal Community State Type (CST) assignment was based on dominant species as identified graphically using HeatMapping : CST I = *Lactobacillus crispatus*, CST II = *Lactobacillus gasseri,* *CST III = Lactobacillus iners*, *CST IV = Low lactobacilli/High Gardnerella vaginalis*, CST V = *Lactobacillus jensenii,* CST VIII = *Bifidobacterium breve,* CST IX = *Streptococcus agalactiae*. Statistical analysis was performed comparing outcomes between CST I, CST III and CST IV. CST groups where n<10 were excluded from analysis given small numbers. Continuous data presented as mean values (SD) for normally distributed data, analysed with one-way ANOVA analysis. * denotes where continuous data was not normally distributed and is expressed as the median with IQR analysed using Kruskal Wallis comparison. Categorical variables are expressed as n, compared using Fishers Exact test .

**Figure S5. Vaginal alpha diversity by Shannon Diversity Index within each Community State Type (CST). Lowest Shannon Diversity Index is seen with CST I and highest is seen in CST IV.**

**
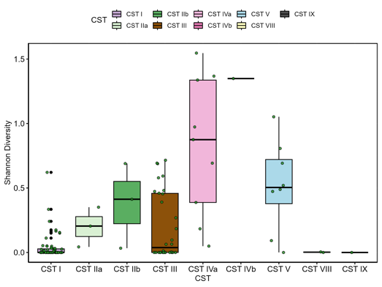
**
